# Association of early pregnancy warm season exposure and neighborhood heat vulnerability with adverse maternal outcomes: A retrospective cohort study

**DOI:** 10.1016/j.joclim.2025.100524

**Published:** 2025-08-19

**Authors:** Melissa Blum, Donato DeIngeniis, Daniela K. Shill, Joanne Stone, Perry Sheffield, Yoko Nomura

**Affiliations:** a Department of Environmental Medicine, Icahn School of Medicine at Mount Sinai, New York, NY, USA; b Department of Psychology, Queens College and The Graduate Center, City University of New York, Queens, NY, USA; c Department of Obstetrics, Gynecology, and Reproductive Science, Icahn School of Medicine at Mount Sinai, New York, NY, USA; d Department of Pediatrics, Icahn School of Medicine at Mount Sinai, New York, NY, USA; e Department of Psychiatry, Icahn School of Medicine at Mount Sinai, New York, NY, USA

**Keywords:** Climate change, Heat, Heat vulnerability, Prenatal exposure, Obstetric complication, Preeclampsia

## Abstract

**Introduction:**

Rising ambient temperatures threaten vulnerable populations such as pregnant women, with urban populations bearing a greater risk due to the urban heat island effect. Here, we assessed the independent effects of trimester-specific warm season exposure during pregnancy and neighborhood heat vulnerability on maternal outcomes, including gestational diabetes, hypertensive disorders of pregnancy, genitourinary infections, and operative delivery.

**Methods:**

This retrospective study analyzed 819 participants from the Stress in Pregnancy Study (2009–2014), a longitudinal birth cohort study in New York City. Generalized linear models examined associations between trimester-specific warm season exposure, New York City Heat Vulnerability Index (ranging 1-5), and adverse maternal outcomes, adjusting for demographics, parity, and substance use.

**Results:**

First trimester warm season exposure was associated with increased odds of gestational hypertension (adjusted odds ratio [AOR] 4.50, 95%CI 1.17-17.27), preeclampsia (AOR 4.38, 95%CI 1.51-12.75), and genitourinary infection (AOR 2.27, 95%CI 1.14-4.51). Each unit increase in heat vulnerability index was associated with increased odds of preeclampsia (AOR 1.38, 95%CI 1.05-1.81) and genitourinary infection (AOR 1.32, 95%CI 1.11-1.57).

**Conclusions:**

Both early pregnancy warm weather exposure and neighborhood vulnerability independently increased the risk of adverse maternal complications. Our findings provide evidence in support of targeted heat mitigation strategies to limit heat exposure in at-risk communities as climate change progresses.

## Highlights

• First trimester heat exposure linked to higher odds of high blood pressure and infection in pregnancy
• Neighborhood heat vulnerability independently raises risk of preeclampsia and infections
• Early pregnancy is a critical window for heat-related perinatal complications
• Study supports targeted heat mitigation to reduce pregnancy risks amid climate change

## Graphical abstract

**Figure:**
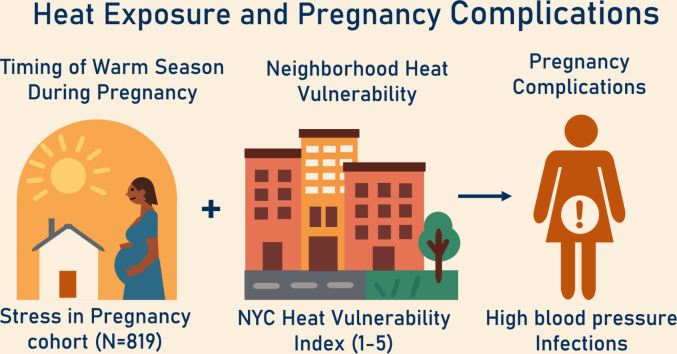

## 1 Introduction

Climate change is increasing ambient temperature and threatening global health and safety, with warming projected to continue through the coming decades. The health risks associated with rising temperatures include dehydration, heat stroke, respiratory disease exacerbations [1], cardiovascular disease [2], and kidney disease [3]. Pregnant women are especially predisposed to poor health outcomes due to physiologic changes that disrupt thermoregulation [4]. Urban residents face added risk from the urban heat island effect, where buildings, roads, and other infrastructure trap heat and reduce nighttime cooling [5].

Evidence suggests that the impact of heat depends on both exposure and vulnerability at multiple levels. Neighborhood characteristics (e.g., high building density, impervious surfaces, and limited greenspace) and individual circumstances (e.g., access to adequate housing and air conditioning) significantly influence heat exposure [6,7]. Various demographic factors (i.e., <18 years old, lower educational attainment, and being a racial or ethnic minority) may also predispose pregnant women to worse health outcomes after heat exposure [8], highlighting the complex interplay between environmental and societal determinants of health.

Pre-existing literature has demonstrated trimester-specific associations between heat exposure and adverse outcomes, suggesting that timing of exposure may be crucial. First trimester heat exposure has been associated with an increased risk of hypertensive disorders of pregnancy [[9], [10], [11]], low birth weight [12], and congenital abnormalities [13], potentially due to perturbations of early organ development and placentation. Second trimester exposure has been linked to gestational diabetes [14,15], possibly reflecting heat-induced metabolic stress during a period of rapid fetal growth. Third trimester heat exposure has been associated with immediate complications and adverse birth outcomes, including vaginal infections [16], low birth weight [17], preterm birth [[17], [18], [19]], and poor markers of newborn health such as low APGAR scores [20,21], highlighting the cumulative effects of heat stress on both maternal and fetal well-being.

Understanding the relationship between heat exposure and pregnancy outcomes is complicated by methodological variations across studies. Researchers have employed various methods to measure heat, ranging from weather station data [9,11] to satellite-derived temperatures [10]. Studies have used these data sources to estimate exposure using ambient temperature thresholds [22], heatwave events [20], average temperature [23], heat indices that incorporate humidity [19], and seasonal categorizations [14]. These varying measurement approaches have made it challenging to establish consensus on the specific timing and duration of heat exposure that confers the greatest risk for different complications. This knowledge gap is particularly significant because pregnancy represents a dynamic period of development, with each trimester featuring distinct physiological and developmental processes that may be differentially affected by heat stress.

To address these knowledge gaps, we conducted a season-based analysis using data from the Stress in Pregnancy (SIP) study in New York City [24]. The SIP study, a longitudinal birth cohort study conducted from 2009-2014, provides a robust framework for examining the association between prenatal stressors, pregnancy complications, and birth outcomes. Our analysis extends the existing work by examining warm season exposure as a trigger of prenatal stress, building on evidence that elevated ambient temperature is associated with high Global Severity Index scores, a metric of stress during pregnancy [25]. By examining the effect of warm season exposure during each trimester, we aim to elucidate the critical heat exposure periods that potentiate pregnancy complications. Importantly, we incorporated the New York City Heat Vulnerability Index, a comprehensive measure of neighborhood-level susceptibility to heat exposure, to examine how structural inequities may independently influence pregnancy outcomes [26]. This multilevel approach allows us to better understand both individual and community-level factors that contribute to heat-related pregnancy risks.

## 2 Methods

### 2.1 Cohort characteristics

We performed a retrospective analysis using data from the Stress in Pregnancy (SIP) study, a longitudinal birth cohort study conducted from 2009-2014 in New York City, which enrolled pregnant women receiving prenatal care at Mount Sinai Hospital or New York Presbyterian-Queens (N = 892) [24]. Exclusion criteria included HIV infection, psychosis, age <15 years old, life-threatening medical complications, congenital or chromosomal abnormalities in the fetus, intrauterine fetal demise, perinatal death, and early withdrawal from the study. While congenital abnormalities are associated with extreme heat [27], the original SIP study was designed to understand the effects of prenatal psychosocial stressors on child neurobehavioral outcomes, which may be confounded by congenital abnormalities, so these participants were excluded from the parent study. The final sample consisted of 819 parent-child dyads (Figure A.1). Apart from race and birth year, there were no significant differences in major demographic variables between participants included versus those excluded from this study (Table A.1). The included group had a higher proportion of White participants and a lower proportion of participants identifying with a race of "Other" compared to the excluded group (X²(3) = 15.26, p<0.001). The included group had a later year of enrollment than the excluded group (X²(5) = 18.78, p<0.001).

### 2.2 Warm season exposure

**Fig. 1:**
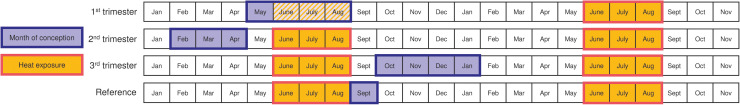
**Schematic of Warm Season Exposure**. Participants were assigned to warm season exposure groups based on which trimester of pregnancy occurred primarily during the warm season months of June, July, and August. For each group, the months of conception are highlighted in purple. The warm season months are highlighted in orange. Months of potential warm season exposure for each group are surrounded by a red box. For the first trimester group, where the warm season overlaps with conception, a striped fill pattern indicates months of overlap.

We defined June, July, and August as the warm season, consistent with meteorological data for New York City during the study period (Figure A.2) [28]. Participants were categorized into four warm season exposure timing groups based on date of conception: first trimester (conception to week 13), second trimester (weeks 14-27), third trimester (week 28 to delivery), or minimal exposure, used as the reference group (Fig. 1). The reference group included pregnancies conceived during September, which largely escaped heat exposure (Table A.2). Date of conception was determined based on available data, using date of birth and gestational age for the majority (78.1%) of participants, followed by estimated date of delivery alone (10.7%), and date of birth alone (11.2%).

### 2.3 New York city heat vulnerability index

We extracted heat vulnerability index scores (range 1-5) using the pregnant participant’s address. The heat vulnerability index incorporates neighborhood poverty rate, racial/ethnic composition, greenspace, access to air conditioning, and surface temperature measurements [26]. Compared to participants with heat vulnerability index < 4, those with heat vulnerability index ≥ 4 lived in neighborhoods with a higher poverty rate, a higher proportion of Non-Hispanic Black residents, less vegetative cover, and less access to air conditioning (Table A.3).

### 2.4 Adverse maternal outcomes

We analyzed all maternal outcomes available from the original SIP cohort data, including pregnancy complications (gestational diabetes mellitus, gestational hypertension, preeclampsia, and genitourinary infection), and mode of delivery (vaginal birth, planned/emergency Cesarean-section). These data were collected at prenatal visits, at delivery by a research nurse, or through electronic medical record review. Gestational diabetes mellitus, gestational hypertension, and preeclampsia were defined per guidelines from the American College of Obstetricians and Gynecologists [29,30]. Genitourinary infection was defined as a new diagnosis with urinary tract infection, abnormal pap smear, HPV, bacterial vaginosis, or a sexually transmitted infection during pregnancy.

### 2.5 Covariates

Models were adjusted for potential confounders, including age, race, ethnicity, marital status, education level, substance use (alcohol, tobacco, and marijuana) during pregnancy, child sex, year, pre-pregnancy weight, and parity. Demographics were based on self-report on enrollment. Race and ethnicity were included as covariates due to accumulating evidence that racism is a driving force behind prenatal health disparities [31]. Participants self-identified as White, Black, Asian, or Other (encompassing American Indian or Alaska Native and Native Hawaiian or Other Pacific Islander) for their race, and Hispanic or Non-Hispanic for their ethnicity. Child sex assigned at birth was recorded by the labor and delivery nurse. Year of pregnancy was defined as the year in which the child was born. Parity and pre-pregnancy weight were collected at prenatal visits or through participants’ self-report.

### 2.6 Statistical analyses

Demographic differences between included and excluded participants, and between the four warm season exposure groups, were examined using analysis of variance for continuous and chi-square tests for categorical variables. The associations between the timing of warm season exposure, heat vulnerability, and maternal outcomes were examined using generalized estimating equations with logit link function nested by family to adjust for intrafamilial correlation between siblings in RStudio, with adjustment for covariates as above. Missing data were imputed using the mice package with 50 imputations [32]. See Table A.4 for missing data frequencies.

### 2.7 Ethical approval

The parent study (SIP study) was approved by the Institutional Review Board at the City University of New York. This secondary analysis was determined exempt from the Program for the Protection of Human Subjects at the Icahn School of Medicine at Mount Sinai (STUDY-23-00250, determined exempt 4/21/2023). All procedures were performed in compliance with the Program for the Protection of Human Subjects at the Icahn School of Medicine at Mount Sinai. The privacy rights of human subjects have been observed and informed consent was obtained from all participants.

## 3 Results

### 3.1 Characteristics of the populations

The study sample was distributed across exposure groups as follows: the reference group with no warm season exposure (n = 97, 11.8%), first trimester exposure (n = 180, 22.0%), second trimester exposure (n = 186, 22.7%), and third trimester exposure (n = 356, 43.5%). There were significant differences in alcohol use (p = 0.03) and year of enrollment (p <0.001) among warm season exposure groups. Full demographic information is shown in Table 1.

**Table 1:** Distribution of Demographic Variables Between Warm Season Exposure Groups.

		**Warm Season Exposure Group**				
	**Total**	**Reference**	**1^st^ TM**	**2^nd^ TM**	**3^rd^ TM**	**Statistics**
**Demographics**	(n = 819)	(n = 97)	(n = 180)	(n = 186)	(n = 356)	
	**Mean (SD)**	**Mean (SD)**	**Mean (SD)**	**Mean (SD)**	**Mean (SD)**	
Maternal age	27.7 (5.8)	28.0 (5.3)	27.2 (5.8)	28.1 (6.0)	27.5 (5.9)	F(3, 815)=0.99, p=0.40
Paternal age	30.5 (7.6)	31.3 (6.9)	29.8 (7.2)	30.9 (7.6)	30.5 (8.0)	F(3, 467)=0.58, p=0.63
Parity	1.80 (1.6)	1.7 (1.7)	2.0 (1.9)	1.9 (1.7)	1.8 (1.6)	F(3, 813)=0.97, p=0.12
Pre-pregnancy weight	151.6 (39.3)	154.7 (45.4)	153.7 (39.8)	152.3 (42.2)	149.4 (35.7)	F(3, 603)=0.56, p=0.64
Heat Vulnerability Index	3.6 (1.6)	3.4 (1.2)	3.4 (1.3)	3.5 (1.3)	3.4 (1.3)	F(3, 723)=0.43, p=0.73
	**N (%)**	**N (%)**	**N (%)**	**N (%)**	**N (%)**	
Race						
White	304 (37.1)	32 (33.0)	66 (36.7)	70 (37.6)	136 (38.2)	
Black	252 (30.8)	26 (26.8)	55 (30.6)	69 (37.1)	102 (28.7)	
Asian	82 (10.0)	14 (14.4)	15 (8.3)	18 (9.7)	35 (9.8)	
Other	181 (22.1)	25 (25.8)	44 (24.4)	29 (15.6)	83 (23.3)	X^2^(9)=11.29, p=0.26
Ethnicity						
Non-Hispanic	381 (46.5)	44 (45.4)	80 (44.4)	96 (51.6)	179 (51.3)	
Hispanic	438 (53.5)	53 (54.6)	100 (55.6)	90 (48.4)	170 (48.7)	X^2^(3)=2.54, p=0.47
Child sex						
Male	423 (51.6)	50 (51.5)	85 (47.2)	98 (52.7)	190 (53.4)	
Female	396 (48.4)	47 (48.5)	95 (52.8)	88 (47.3)	166 (46.6)	X^2^(3)=1.92, p=0.59
Marital status						
Married	320 (39.3)	37 (38.1)	64 (35.6)	84 (45.4)	131 (38.2)	
Common Law	54 (6.6)	7 (7.2)	12 (6.7)	7 (3.8)	28 (7.9)	
Single	416 (51.0)	48 (49.5)	100 (55.6)	87 (47.0)	177 (51.3)	
Divorced/Separated	3 (0.4)	0 (0)	0 (0)	1 (0.5)	2 (0.6)	
Widowed	22 (2.7)	5 (5.2)	4 (2.2)	6 (3.2)	8 (2.0)	X^2^(12)=11.84, p=0.46^1^
Education						
Primary school	20 (2.5)	2 (2.1)	2 (1.1)	7 (3.8)	9 (2.5)	
Some high school	106 (13.0)	9 (9.3)	30 (16.7)	22 (11.9)	45 (12.7)	
High school/GED	197 (24.1)	26 (26.8)	47 (26.1)	37 (20.0)	86 (24.4)	
Some college	211 (25.9)	28 (28.9)	46 (25.6)	45 (24.3)	92 (26.1)	
Associate’s degree	87 (10.7)	9 (9.3)	20 (11.1)	18 (9.7)	40 (11.3)	
Bachelor’s degree	109 (13.4)	15 (15.5)	17 (9.4)	33 (17.8)	44 (12.5)	
Graduate degree	86 (10.5)	8 (8.2)	18 (10.0)	23 (12.4)	37 (10.5)	X^2^(18)=15.27, p=0.64
Tobacco						
Yes	39 (5.7)	7 (9.3)	5 (3.2)	7 (4.0)	20 (7.0)	
No	650 (94.3)	68 (90.7)	149 (96.8)	166 (96.0)	267 (93.0)	X^2^(3)=5.34, p=0.15
Marijuana						
Yes	35 (5.1)	0 (0)	7 (4.6)	11 (6.4)	17 (5.9)	
No	654 (94.9)	75 (100)	146 (95.4)	162 (93.6)	271 (94.1)	X^2^(3)=5.09, p=0.11^1^
Alcohol						
Yes	39 (5.7)	0 (0)	14 (9.2)	9 (5.2)	16 (5.6)	
No	650 (94.3)	75 (100)	139 (90.8)	164 (94.8)	272 (94.4)	X^2^(3)=8.06, p=0.03^1^
Year of birth						
2010	132 (16.1)	17 (17.5)	7 (3.9)	26 (14.0)	82 (23.0)	
2011	116 (14.2)	10 (10.3)	18 (10.0)	44 (23.7)	44 (12.4)	
2012	208 (25.4)	19 (19.6)	77 (42.8)	40 (21.5)	72 (20.2)	
2013	274 (33.5)	42 (43.3)	61 (33.9)	47 (25.3)	124 (34.8)	
2014	70 (8.5)	6 (6.2)	16 (8.9)	24 (12.9)	24 (6.7)	
2015	19 (2.3)	3 (3.1)	1 (0.6)	5 (2.7)	10 (2.8)	X^2^(15) = 87.38, p<0.001^1^

1 Fisher’s exact test was used. The number and percentage of participants in each demographic category are shown. For continuous covariates, descriptive statistics are shown. N may vary due to missing values. 1^st^ TM = first trimester exposure; 2^nd^ TM = second trimester exposure; 3^rd^ TM = third trimester exposure. NE = not estimable. N may vary due to missing data.

### 3.2 Association of trimester-specific heat exposure with maternal complications

Trimester-specific heat exposure and heat vulnerability index showed distinct associations with maternal complications. First trimester warm season exposure was associated with increased odds of gestational hypertension (adjusted odds ratio (AOR) 4.50, 95%CI 1.17, 17.27), preeclampsia (AOR 4.38, 95%CI 1.51, 12.75), and genitourinary infection (AOR 2.27, 95%CI 1.14, 4.51) (Table 2). Third trimester warm season exposure showed a trend of increased odds of preeclampsia (AOR 2.69, 95%CI 0.94, 7.72).

**Table 2:** Association of Trimester of Warm Season Exposure, Maternal Complications, and Mode of Delivery.

**Maternal Complication**	**N (%) affected**	**Unadjusted**		**Adjusted**	
		**OR (95%CI)**	***p***	**AOR (95%CI)**	***p***
Gestational Diabetes					
Reference	8 (8.4)	1.00		1.00	
1^st^ TM	33 (18.3)	2.37 (0.97, 5.78)	0.06	1.92 (0.85, 4.33)	0.12
2^nd^ TM	15 (8.1)	0.94 (0.36, 2.46)	0.90	0.88 (0.36, 2.17)	0.79
3^rd^ TM	23 (7.4)	0.93 (0.38, 2.28)	0.87	1.07 (0.47, 2.43)	0.87
Gestational Hypertension					
Reference	3 (3.2)	1.00		1.00	
1^st^ TM	19 (10.7)	3.83 (1.10, 13.27)	0.03	4.50 (1.17, 17.27)	0.03
2^nd^ TM	12 (6.5)	2.24 (0.60, 8.29)	0.30	2.37 (0.53, 10.67)	0.26
3^rd^ TM	14 (4.5)	1.59 (0.43, 5.87)	0.49	1.91 (0.46, 7.88)	0.37
Preeclampsia					
Reference	5 (5.3)	1.00		1.00	
1^st^ TM	30 (16.9)	3.60 (1.34, 9.68)	0.01	4.38 (1.51, 12.75)	0.006
2^nd^ TM	16 (8.6)	1.69 (0.59, 4.87)	0.33	1.61 (0.51, 5.07)	0.41
3^rd^ TM	37 (11.7)	2.44 (0.92, 6.46)	0.07	2.69 (0.94, 7.72)	0.07
Genitourinary Infection					
Reference	16 (16.5)	1.00		1.00	
1^st^ TM	47 (26.4)	1.80 (0.95, 3.40)	0.07	2.27 (1.14, 4.51)	0.02
2^nd^ TM	35 (19.0)	1.19 (0.62, 2.29)	0.60	1.22 (0.60, 2.51)	0.58
3^rd^ TM	76 (22.2)	1.40 (0.77, 2.54)	0.27	1.39 (0.74, 2.62)	0.31
Emergency C-section					
Reference	6 (6.2)	1.00		1.00	
1^st^ TM	6 (3.4)	0.56 (0.19, 1.63)	0.29	0.63 (0.21, 1.85)	0.39
2^nd^ TM	12 (6.5)	1.05 (0.37, 2.96)	0.93	1.31 (0.44, 3.88)	0.63
3^rd^ TM	25 (7.0)	1.15 (0.45, 2.90)	0.77	1.37 (0.54, 3.47)	0.51
Planned C-section					
Reference	16 (16.5)	1.00		1.00	
1^st^ TM	32 (18.0)	1.10 (0.57, 2.14)	0.77	1.08 (0.54, 2.19)	0.36
2^nd^ TM	50 (27.0)	1.88 (1.00, 3.55)	0.05	1.82 (0.95, 3.50)	0.33
3^rd^ TM	68 (19.2)	1.20 (0.66, 2.18)	0.55	1.19 (0.64, 2.19)	0.31

Reference = pregnancy conceived in September (N=97); 1^st^ TM = first trimester occurred primarily during the warm season (June, July, August) (N=180); 2^nd^ TM = second trimester occurred primarily during the warm season (N=186); and 3^rd^ TM = third trimester occurred primarily during the warm season (N=356). OR = odds ratio; AOR = adjusted odds ratio; CI = confidence interval. The adjusted model includes the following as covariates for statistical adjustment: individual-level demographic factors (age, marital status, educational level, race, ethnicity, and child sex), risk factors for pregnancy complications (tobacco, marijuana, alcohol, and pre-pregnancy weight, and parity (# of times a person has given birth to a fetus with a gestational age >24 weeks)), neighborhood-level risk factors for heat exposure represented by the New York City heat vulnerability index, and year.

### 3.3 Association of heat vulnerability index with maternal complications

Independent of warm season exposure timing, each unit increase in heat vulnerability index (range 1-5) was associated with increased odds of preeclampsia (AOR 1.32, 95%CI 1.06, 1.64) and genitourinary infection (AOR 1.29, 95%CI 1.08, 1.53) (Table 3). We observed no associations between trimester of warm season exposure or heat vulnerability index with either gestational diabetes mellitus or mode of delivery. The incidence of maternal complications in participants with both first trimester warm season exposure and high heat vulnerability index scores is show in Table 4.

**Table 3:** Association of heat vulnerability index and maternal complications.

**Maternal Complication**	**Unadjusted**		**Adjusted**	
	**OR (95%CI)**	***p***	**AOR (95%CI)**	***p***
Gestational Diabetes	1.04 (0.84, 1.28)	0.74	1.14 (0.90, 1.44)	0.28
Gestational Hypertension	1.11 (0.89, 1.38)	0.37	1.22 (0.94, 1.59)	0.14
Preeclampsia	1.41 (1.18, 1.70)	<0.001	1.32 (1.06, 1.64)	0.01
Genitourinary Infection	1.36 (1.20, 1.55)	<0.001	1.29 (1.08, 1.53)	0.005
Emergency C-section	0.79 (0.63, 0.98)	0.03	0.83 (0.64, 1.09)	0.18
Planned C-section	1.05 (0.94, 1.19)	0.39	1.04 (0.89, 1.22)	0.62

OR = odds ratio; AOR = adjusted odds ratio; CI = confidence interval. The adjusted model includes the following as covariates for statistical adjustment: individual-level demographic factors (age, marital status, educational level, race, ethnicity, and child sex), risk factors for pregnancy complications (tobacco, marijuana, alcohol, and pre-pregnancy weight, and parity (# of times a person has given birth to a fetus with a gestational age >24 weeks)), trimester of warm season exposure, and year. Heat vulnerability index was examined as a continuous outcome.

**Table 4:** Incidence of maternal complications with warm season exposure and high vs. low heat vulnerability index.

**Maternal Complication**	**Number and Percentage Affected (N(%))**	
	**Low HVI + 1st TM Exposure (N = 73)**	**High HVI + 1st TM Exposure (N = 78)**
Gestational Diabetes	9 (12.3)	13 (16.7)
Gestational Hypertension	6 (8.2)	9 (11.5)
Preeclampsia	8 (11)	15 (19.2)
Genitourinary Infection	17 (23.3)	29 (37.2)
Emergency C-section	4 (5.6)	1 (1.3)
Planned C-section	12 (16.7)	15 (19.2)

1^st^ TM = first trimester occurred primarily during the warm season (June, July, August); High HVI = participants residing in a neighborhood with heat vulnerability index ≥ 4; Low HVI = participants residing in a neighborhood with heat vulnerability index < 4. The number and percentage of participants with first trimester warm season exposure who were affected by maternal complications is shown, subdivided by high vs. low heat vulnerability index.

## 4 Discussion

### 4.1 Key findings

The study has two major findings that highlight both individual and neighborhood-level impacts of heat exposure during pregnancy. First, we identified associations between early pregnancy warm season exposure and increased odds of several common pregnancy complications, including gestational hypertension, preeclampsia, and genitourinary infections. Second, neighborhood-level risk factors represented by heat vulnerability index were independently associated with increased odds of preeclampsia and genitourinary infection, pointing to the role of structural and environmental inequities in shaping pregnancy outcomes.

### 4.2 Contribution to the literature

Our study expands on existing literature by evaluating critical periods for heat exposure in a diverse urban cohort and adjusting for a broad array of individual-level demographics and obstetric risk factors to minimize unmeasured confounding. Our findings suggest that the first trimester is a critical window of vulnerability during which warm seasonal conditions can have profound effects on maternal and fetal health, corroborating prior studies showing elevated risk of hypertensive disorders of pregnancy following heat exposure in the first 20 weeks of pregnancy [33]. The observed relationship between third trimester warm season exposure and preeclampsia may reflect a previously reported second window of susceptibility later in pregnancy [34]. Warm season exposure during the first trimester may impair the establishment of healthy vascular exchange, producing hypertension later in pregnancy due to placental oxygen insufficiency and release of vasoconstrictive mediators when rapid fetal growth requires accelerated nutrient transfer [35]. Heat exposure may also upregulate heat shock proteins in response to endoplasmic reticulum stress from heat-induced protein misfolding. These proteins are highly expressed in endometrial and placental cells, and their dysfunction may disrupt placental development and contribute to hypertension [36].

The observed association between first trimester warm season and genitourinary infection contributes to limited literature on seasonal patterns of antepartum infections. Prior studies have primarily documented increased group B strep colonization late in pregnancy during warmer periods [16]. Studies have shown increased incidence of urinary tract infections during periods of higher ambient temperature and variation in the dominant strains by season, though none have specifically examined seasonal variation of urinary tract infections during pregnancy [37,38]. One study noted increased rates of sexually transmitted infections during the summer and fall, possibly due to seasonal patterns of sexual activity [39]. While we cannot assess the latency between first trimester heat exposure and the infection onset, future studies could clarify the kinetics and mechanism. Given the risks of ascending or vertically transmitted infections for both the pregnant person and the fetus, identifying pathogens with strong seasonal patterns remains a priority.

Of note, we did not identify any associations between trimester of warm season exposure and gestational diabetes mellitus or mode of delivery, despite previously reported associations between second trimester heat exposure and gestational diabetes mellitus [14,15,23]. Specific heat events or temperature variability within seasons, which are not captured by our season-based approach, may be more likely to drive changes in the risk of gestational diabetes mellitus. Investigations of lifestyle factors such as diet and physical activity, which were not included in this study, could also help to clarify this finding in future studies. While we observed an increase in hypertensive disorders of pregnancy and genitourinary infections in the first trimester group, these complications may have been managed without the need for urgent interventions such as emergency C-section, resulting in no differences in the mode of delivery overall.

We examined the interplay of environmental exposures and pre-existing socioeconomic vulnerabilities by incorporating heat vulnerability index, which includes important protective factors such as greenspace and access to air conditioning that may safeguard later life wellbeing in the setting of prenatal heat exposure [40]. Independently of warm season exposure, elevated heat vulnerability at the neighborhood level was a risk factor for both preeclampsia and genitourinary infection, suggesting that neighborhood-level socioeconomic factors are a key determinant of maternal health outcomes. At the individual level, abundant evidence correlates race and socioeconomic status with pregnancy complications such as preeclampsia, with Black and African American women consistently experiencing higher incidence, morbidity, and mortality [41,42]. Moreover, recent studies suggest a role for neighborhood composition and individual level socioeconomic status in modifying the vaginal microbiome [43], which could contribute to the risk of genitourinary infections. Prior studies have shown a protective effect of greenspace in reducing the risk of mood disorders during pregnancy and gestational diabetes, although a similar effect was not shown for hypertensive disorders of pregnancy [44]. These findings underscore the critical role of neighborhood-level factors in shaping maternal health risks, highlighting the need for targeted interventions to mitigate disparities.

### 4.3 Limitations

The study has several limitations that necessitate cautious interpretation of the findings. First, although validated by meteorological data, our season-based approach offers a less precise modality for quantifying heat exposure and does not account for temperature variability within seasons due to extreme heat events. Given the variety of heat exposure approaches previously employed in the literature, we selected an approach that is broadly applicable to temperate zones and does not give a false sense of precision, unlike approaches using meteorological data which may be distorted by protective factors such as air conditioner use and indoor employment. Second, the generalizability of our findings may be compromised due to changing conditions over time, such as increased heat events and shifting neighborhood demographics since our data were collected. However, meteorological data consistently show temperatures are rising during the warm season while wealth inequality and socioeconomic segregation persist in New York City, suggesting that impacts may be even greater today. Third, due to limited sample size, we were only able to examine genitourinary infections in aggregate, although individual pathogens may have unique associations with warm season exposure. Fourth, an uneven recruitment pace resulted in disproportionate numbers of participants across exposure groups. Fifth, despite adjusting for race and year of pregnancy, application of the exclusion criteria may have biased the associations, as there were significant differences in race and year of pregnancy between included and excluded participants (Table A.1).

Finally, while we explored neighborhood-level effects, our sample size limited our ability to conduct robust analyses of how individual-level factors (race, ethnicity, socioeconomic status, and access to heat mitigation strategies) might independently influence maternal outcomes, and how these factors interact with neighborhood-level exposures. Power analyses revealed that our study had insufficient statistical power (<15%) to detect small interaction effects between trimester-specific warm season exposure and heat vulnerability (Table A.5). The cohort was also underpowered to explore the relationship between warm season exposure and adverse fetal outcomes known to be associated with heat exposure, including stillbirth [45]. Future research with larger, more diverse cohorts would be better positioned to investigate these critical interactions and identify specific populations who face compounded vulnerability from both individual and neighborhood-level risk factors. Given the emerging climate crisis and its potential impact on maternal health, we prioritized reporting these preliminary findings while acknowledging that replication in larger cohorts will be essential to confirm the patterns observed.

## 5 Conclusions

Because prenatal complications may predict future health for pregnant women [46] and predispose offspring to later life challenges [47], our findings call for both improved patient education to communicate the risks of prenatal warm season exposure and investment in removing structural barriers which can endanger communities. The continuing threat of climate change makes this area of research an urgent public health priority, particularly as the intensity and frequency of heat events are projected to increase in coming decades and the United States lags behind peer nations in pregnancy outcomes.

The associations between neighborhood heat vulnerability and adverse maternal outcomes documented in this study may also lead to worsening disparities in maternal health across socioeconomic and racial lines without clinical and public health intervention. Our findings suggest that clinicians should consider both seasonal timing and neighborhood vulnerability when assessing risk during prenatal care, particularly for patients in their first trimester during the summer months. Healthcare systems serving heat-vulnerable communities may need to implement enhanced patient education about heat-related risks, along with targeted interventions such as expanded cooling center access for pregnant women and heat warning systems specific to maternal health. These strategies can help mitigate climate-related risks and advance equity in maternal outcomes.

## CRediT authorship contribution statement

**Melissa Blum:** Writing – review & editing, Writing – original draft, Visualization, Validation, Formal analysis, Data curation, Conceptualization. **Donato DeIngeniis:** Writing – review & editing, Formal analysis, Data curation, Conceptualization. **Daniela K. Shill:** Writing – review & editing. **Joanne Stone:** Writing – review & editing. **Perry Sheffield:** Writing – review & editing, Supervision, Resources, Funding acquisition. **Yoko Nomura:** Writing – review & editing, Supervision, Software, Resources, Funding acquisition, Data curation, Conceptualization.

## Declaration of competing interest

The authors declare that they have no known competing financial interests or personal relationships that could have appeared to influence the work reported in this paper.

## Appendix Supplementary materials

Image, application 1
