# Supplementary material for: Association of early pregnancy warm season exposure and neighborhood heat vulnerability with adverse maternal outcomes: A retrospective cohort study
Source: J Clim Chang Health. 2025 Aug 19;25:100524. doi: 10.1016/j.joclim.2025.100524 (PMC12525232; doi:10.1016/j.joclim.2025.100524)
Supplement: Supplementary file 1 [file mmc1.docx]

**Appendix A**

**Figure A.1.** **Flow chart of participants included in the study**

Stress in Pregnancy Study

Pregnant participants: n = 892

Excluded

- Ineligible (n = 28)

Eligible

Pregnant participants: n = 864 (96.86%)

Excluded

- Pregnancy loss (n = 19)

Included

Pregnant participants: n = 819 (96.92%)

Included

Pregnant participants: n = 845 (97.80%)

- Moved out of NY (n = 12)
- Withdrawn (n = 14)

Flow chart of participants excluded due to ineligibility, pregnancy loss, moving out of the New York area, or withdrawal from the study.

**Figure A.2. Validation of June through August as the Warm Season in New York City**

**
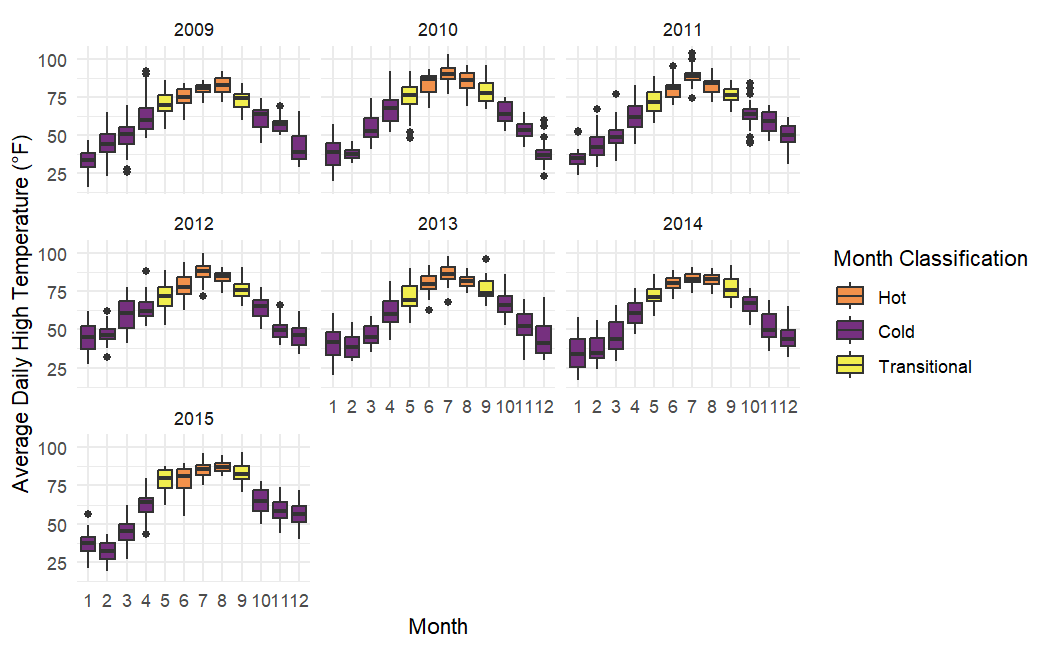
**

Publicly available weather data for Central Park (station ID USW00094728) was extracted from the Climate Data Online portal maintained by the National Centers for Environmental Information. The average daily high temperature at the Central Park weather station for each month during the study period (2009-2015) is shown above. The hot months of June, July, and August were consistently warmer on average than the transitional months of May and September and the cold months of January, February, March, April, October, November, and December, with one exception. In 2015, September was warmer on average than June. However, only 5 participants in our study had dates of birth in September 2015 or later, so our selection of June through August as the warmest months is largely supported for our cohort.

**Table A.1.** **Characteristics of participants included and not included in the study**

|  | **Included** | **Not included** |  |
| --- | --- | --- | --- |
| **Demographics** | **(n = 819)** | **(n = 45)** | **Statistics p-value** |
|  | **Mean (SD)** | **Mean (SD)** |  |
| Maternal age | 27.7 (5.8) | 27.1 (6.0) | F(1, 829) = 0.09, p=.76 |
| Paternal age | 30.5 (7.6) | -- | NE |
| Parity | 1.80 (1.6) | 2.4 (2.3) | F(1, 825) = 2.99, p=.08 |
| Pre-pregnancy weight (lb) | 151.6 (39.3) | 149.8 (31.4) | F(1, 609) < 0.01, p=.99 |
| Heat vulnerability index | 3.6 (1.6) | 3.6 (1.6) | F(1, 757) = 0.24, p=.62 |
|  | **N (%)** | **N (%)** |  |
| Race |  |  |  |
| White | 304 (37.1) | 8 (13.6) |  |
| Black | 252 (30.8) | 20 (33.9) |  |
| Asian | 82 (10.0) | 3 (5.1) |  |
| Other | 181 (22.1) | 28 (47.5) | X^2^(3) = 15.26, p<.001^1^ |
| Ethnicity |  |  |  |
| Non-Hispanic | 381 (46.5) | 29 (49.2) |  |
| Hispanic | 438 (53.5) | 30 (50.8) | X^2^(1) = 0.88, p=.35 |
| Child sex |  |  |  |
| Male | 423 (51.6) | 10 (38.5) |  |
| Female | 396 (48.4) | 16 (61.5) | X^2^(1) = 1.75, p=.19 |
| Marital status |  |  |  |
| Married | 320 (39.3) | 6 (25.0) |  |
| Common Law | 54 (6.6) | 1 (4.2) |  |
| Single | 416 (51.0) | 17 (70.8) |  |
| Widowed | 3 (0.4) | 0 |  |
| Divorced/Separated | 22 (2.7) | 0 | X^2^(4) = 3.94, p=.41^1^ |
| Education |  |  |  |
| Primary School | 20 (2.5) | 0 |  |
| Some high school | 106 (13.0) | 5 (20.8) |  |
| High School/GED | 197 (24.0) | 10 (41.7) |  |
| Some College | 211 (25.9) | 7 (29.2) |  |
| Associate’s degree (2-year college) | 87 (10.7) | 0 |  |
| Bachelor’s degree (4-year college) | 109 (13.4) | 1 (4.2) |  |
| Graduate/professional degree | 86 (10.6) | 1 (4.2) | X^2^(6) = 9.70, p=.16^1^ |
| Tobacco |  |  |  |
| Yes | 39 (5.7) | 0 (0) |  |
| No | 650 (94.3) | 10 (100) | X^2^(1) = 0.60, p>.99^1^ |
| Marijuana |  |  |  |
| Yes | 35 (5.1) | 1 (10.0) |  |
| No | 654 (94.9) | 9 (90.0) | X^2^(1) = 0.49, p=.41^1^ |
| Alcohol |  |  |  |
| Yes | 39 (5.7) | 0 (0) |  |
| No | 650 (94.3) | 10 (100) | X^2^(1) = 0.60, p>.99^1^ |
| Year |  |  |  |
| 2010 | 132 (16.1) | 9 (36.0) |  |
| 2011 | 116 (14.2) | 1 (4.0) |  |
| 2012 | 208 (25.4) | 10 (40.0) |  |
| 2013 | 274 (33.5) | 5 (20.0) |  |
| 2014 | 70 (8.5) | 0 (0) |  |
| 2015 | 19 (2.3) | 0 (0) | X^2^(5) = 18.78, p<.001^1^ |

^1^Fisher’s exact test was used.

The number and percentage of participants in each demographic category are shown. For continuous covariates, descriptive statistics are shown. N may vary due to missing values. 1^st^ TM = first trimester exposure; 2^nd^ TM = second trimester exposure; 3^rd^ TM = third trimester exposure; NE = not estimable; GED = general educational development

**Table A.2. Validation of Warm Season Exposure Group Assignment**

| **Group** | | **Average Daily High Temperature (°F)** | | | | **Number of Days Over 90°F** | | | |
| --- | --- | --- | --- | --- | --- | --- | --- | --- | --- |
|  |  | Mean | SD | β | p | Mean | SD | β | p |
| **Overall cohort** | | 63.1 | 5.3 | -- | -- | 14.9 | 9.6 | -- | -- |
| **Trimester of Warm Season Exposure** | Reference | 56.6 | 1.9 | -- | -- | 2.6 | 3 | -- | -- |
|  | 1^st^ TM | 60.2 | 3.4 | 3.5 | <0.001 | 8.7 | 7.1 | 6.2 | <0.001 |
|  | 2^nd^ TM | 69.3 | 2.7 | 12.7 | <0.001 | 20.4 | 6.9 | 17.8 | <0.001 |
|  | 3^rd^ TM | 63.1 | 4.4 | 6.5 | <0.001 | 18.4 | 8.2 | 15.9 | <0.001 |

We selected Central Park and LaGuardia Airport weather stations as they are the closest weather stations to the sites where participants were recruited for the study (Mount Sinai Hospital and New York Presbyterian-Queens). For each participant, we used the weather station closest to their hospital of enrollment in the study to calculate average daily high temperature and the number of days with a high temperature exceeding 90°F that occurred during pregnancy. The threshold temperature of 90°F was chosen to represent exposure to extreme heat because exposure to ambient temperatures above this threshold have been shown to reduce placental blood flow.[1] The reference group experienced a lower average daily high temperature and fewer days over 90°F than all other warm season exposure groups, validating our system for assignment of participants. Ref = conceived in September, minimal warm season exposure; 1^st^ TM = primarily exposed to the warm season during the first trimester; 2^nd^ TM = primarily exposed to the warm season during the second trimester; 3^rd^ TM = primarily exposed to the warm season during the third trimester.

**Table A.3. Missing Data Frequencies**

| **Variable** | **Number missing** | **Percentage Missing (%)** |
| --- | --- | --- |
| Trimester of Warm Season Exposure | 0 | 0.0 |
| Gestational Diabetes | 49 | 6.0 |
| Gestational Hypertension | 48 | 5.9 |
| Preeclampsia | 47 | 5.7 |
| Genitourinary Infection | 18 | 2.2 |
| Emergency C-section | 4 | 0.5 |
| Planned C-section | 4 | 0.5 |
| Year | 0 | 0.0 |
| Pre-pregnancy weight | 212 | 25.9 |
| Child sex | 0 | 0.0 |
| Age of pregnant person | 0 | 0.0 |
| Marital status | 4 | 0.5 |
| Educational attainment | 4 | 0.5 |
| Race | 0 | 0.0 |
| Ethnicity | 0 | 0.0 |
| Parity | 2 | 0.2 |
| Prenatal marijuana use | 130 | 15.9 |
| Prenatal tobacco use | 130 | 15.9 |
| Prenatal alcohol use | 130 | 15.9 |
| Heat vulnerability index | 89 | 10.9 |

The frequency of missing data for each variable is shown above.

**Table A.4. Power Analysis: Interaction of Warm Season Exposure and Heat Vulnerability**

| **Interaction Term** | **Effect Size** | **Power** |
| --- | --- | --- |
| 1^st^ TM * HVI | 0.15 | 0.15 |
| 2^nd^ TM * HVI | 0.10 | 0.11 |
| 3^rd^ TM * HVI | 0.05 | 0.07 |

Power was estimated via simulation using the lme4 package in RStudio with 100 simulations and an N of 819[2]. Simulations assumed a 10% prevalence of the outcome and an alpha of 0.05. As there is no pre-existing literature examining this interaction, we assumed a small effect size to be conservative. The statistical power to detect the above effect sizes was less than 80%. 1^st^ TM = first trimester, 2^nd^ TM = second trimester, 3^rd^ TM = third trimester, HVI = New York City Heat Vulnerability Index.

**References**

[1] Bonell A, Vannevel V, Sonko B, Mohammed N, Vicedo-Cabrera AM, Haines A, et al. A feasibility study of the use of UmbiFlow™ to assess the impact of heat stress on fetoplacental blood flow in field studies. Int J Gynaecol Obstet. 2023;160:430-6.

[2] Bates D, Mächler M, Bolker B, Walker S. Fitting Linear Mixed-Effects Models Using lme4. Journal of Statistical Software. 2015;67:1 - 48.
